# Secretory Leukocyte Protease Inhibitor Is an Inducible Antimicrobial Peptide Expressed in *Staphylococcus aureus* Endophthalmitis

**DOI:** 10.1155/2007/93857

**Published:** 2007-12-03

**Authors:** Victor E. Reviglio, Ruben H. Sambuelli, Alejandra Olmedo, Micaela Falco, Jose Echenique, Terrence P. O'Brien, Irene C. Kuo

**Affiliations:** ^1^Cornea and External Eye Diseases Lab, School of Medicine, Catholic University of Cordoba, Cordoba 5021, Argentina; ^2^Department of Biochemistry and Molecular Biology, School of Medicine, National University of Cordoba, Cordoba 5022, Argentina; ^3^Cornea and External Diseases, Bascom Palmer, School of Medicine, University of Miami, Miami, FL 33136, USA; ^4^Cornea Department, The Wilmer Eye Institute, Johns Hopkins University, Baltimore, MD 21287, USA

## Abstract

*Purpose*. To describe the presence of secretory leukocyte protease inhibitor (SLPI), a cationic peptide with antimicrobial and antiprotease activity, in the innate ocular immune reaction in a rat model of *Staphylococcus aureus* endophthalmitis. *Methods*. Seventy-five female Lewis rats were divided into three groups: the endophthalmitis group received an intravitreal injection of 65 colony-forming units of viable *S. aureus*, the vehicle-injected group received balanced sterile saline solution (BSS), and the control group was not injected. Eyes were enucleated at 24 and 48 hours and processed for immunohistochemical staining and Western blot studies for SLPI. *Results*. In *S. aureus* endophthalmitis eyes, there was strong immunostaining for SLPI in the retina and vitreous with associated neutrophilic infiltrates. At 48 hours, corneas also stained for SLPI. Western blots confirmed increased SLPI expression in all infected eyes. By immunohistochemical assays, SLPI was absent in the BSS and control eyes. The causative pathogen was identified in all samples from the endophthalmitis group by traditional culture methods. *Conclusions*. To our knowledge, this report is the first to demonstrate the presence of SLPI in the inflamed cornea, vitreous, and retina tissues of rat eyes with *S. aureus* endophthalmitis, suggesting that SLPI has an active role in the innate immunity of the eye. Release of SLPI by inflammatory cells in the anterior and posterior segments may contribute to the host defense response against infectious endophthalmitis.

## 1. INTRODUCTION

Infectious endophthalmitis is a potentially devastating complication of intraocular surgery, most commonly cataract extraction [1, 2]. Within hours, tissue damage and consequent loss of vision result from the inflammatory process [2]. *Staphylococcus epidermidis* and *Staphylococcus aureus* have remained the most prevalent infectious agents in postoperative bacterial endophthalmitis [3]. Early diagnosis and prompt treatment are essential to allay the host inflammatory response [1]. Specific and nonspecific defense mechanisms play an important role in ocular immunity, maintaining a delicate balance between effective defenses and potentially harmful inflammation responses [4]. Antimicrobial peptides contribute to innate immune defense against a number of Gram positive and Gram negative bacteria, viruses, and fungi [5]. These peptides include secretory leukocyte protease inhibitor (SLPI) [6–8], a cationic peptide, as well as defensins and cathelicidins.

Human SLPI is an 11.7 kDa nonglycosylated protein initially isolated from respiratory mucosal epithelial cells [8]. It is composed of two domains: a protease inhibitor at the carboxyl-terminal domain and the antimicrobial amino-terminal domain [8–10]. SLPI has defensin-like antibacterial activities and suppresses the production of inflammatory mediators [9]. Recent studies demonstrate that macrophages secrete SLPI in response to bacterial lipopolysaccharides and toxins; therefore, we assume that SLPI modulates the ocular immune response in endophthalmitis [11, 12]. 

To determine whether SLPI has a role in inflammation and infection of the eye, where SLPI has not been described before, we investigated and quantified SLPI expression in normal and infected ocular tissues using a murine bacterial endophthalmitis model.

## 2. MATERIALS AND METHODS

### 2.1. Experimental design

Animals were handled in compliance with the tenets of the Association for Research in Vision and Ophthalmology (ARVO) statement for the use of animals in Ophthalmic and Vision Research and the Guide for the Care and Use of Laboratory Animals (National Research Council). All experiments were approved by the Institutional Animal Care Committee of the Catholic University of Cordoba, Argentina.

Seventy-five female Lewis rats, each weighing 250 g, were divided amongst three groups: (1) the *S. aureus* inoculated group (30 rats), (2) the vehicle-injected group (30 rats), and (3) the un-injected control group (10 rats). The right eye of the *S. aureus* and the vehicle-injected rats received intravitreal injections of *S. aureus* inoculum and balanced salt solution (BSS), respectively; the left eye was uninjected. The rats were divided as follows: of the 30 rats in the *S. aureus* group, 8 rats were assigned to immunohistochemistry at 24 hours, 7 rats to Western blotting at 24 hours, 8 rats to immunohistochemistry at 48 hours, and 7 rats to Western blotting at 48 hours. The same was done with the 30 rats in the vehicle-injected group. The 10 rats without injection were divided into 5 rats for immunohistochemistry and 5 rats for Western blotting.

*S. aureus* from a human endophthalmitis sample was cultured in tryptase soy broth. The bacterial suspension was centrifuged, and washed with sterile saline. The suspension was serially diluted with sterile saline to 65 CFU/50 *μ*L. Each rat was anesthetized with an intramuscular injection of 0.125 ml of a 1 : 1 mixture of 100 mg/ml ketamine and 20 mg/ml xylazine; a drop of proparacaine 0.5% was instilled in the right eye of *S. aureus* inoculated and sham injected rats. Five *μ*L of aqueous humor was aspirated from the experimental eyes to minimize any increase in intraocular pressure with the subsequent inoculation of *S. aureus* or BSS. The experimental *S. aureus* group received an intravitreal injection of 50 *μ*L (65 CFU) of *S. aureus* suspension through the pars plana, and the vehicle-injected group received 50 *μ*L of BSS. Postinjection eyes were irrigated with BSS. Rats were euthanized using phenobarbital at 24 or 48 hours after the injection, and eyes were harvested for immunohistochemical studies.

### 2.2. Fixation and processing of tissue for immunohistochemistry

The right eyes from the *S. aureus* inoculated (eight rats for each time point), vehicle-injected groups (eight rats for each time point), and untreated control (5 rats) groups euthanized at 24 and 48 hours were enucleated for immunohistochemical studies. The eyes were submerged in 10% buffered formalin for 3 days, washed with distilled water, rehydrated through a graded series of ethanol, embedded in paraffin, and processed for immunohistochemistry.

Immunohistochemical staining was performed using an avidin-biotin-peroxidase complex technique. Paraffin-embedded sections were treated with 0.6% hydrogen peroxide in methanol and blocked with 10% normal goat serum. Primary antibody consisted of 1 : 100 dilution of polyclonal goat anti-SLPI at 1 : 100 dilution (Santa Cruz Biotechnology, Inc., Santa Cruz, Calif, USA) was applied to the eye sections, incubated at room temperature for 1 hour, and the unbound antibody was removed with TBS (20 mM Tris-HCl pH 7.5, 150 mM NaCl). A biotinylated rabbit anti-goat IgG secondary antibody (Vector Laboratories, Burlingame, Calif, USA) was applied and amplified with avidin-biotin-peroxidase complex (Vector Laboratories, Burlingame, Calif, USA). Signals were developed for visualization with 3,3′ diaminobenzidine. Control sections were incubated with normal goat serum. All samples were stained in parallel to minimize specimen variation. Masked pathologists graded the cell staining intensity quantitatively.

### 2.3. Western blot analysis

Vitreous samples were collected from the right eyes of seven rats from the *S. aureus* inoculated group, seven rats from the vehicle-injected group, and 5 rats from the control group using a 20-gauge needle. Retina tissue was excised under a dissecting microscope by masked pathologist and placed in a sterile tube. Vitreous samples and retina tissue were homogenized separately in phosphate-buffered saline with 100 **μ**M butylated-hydroxytoluene and centrifuged for 10 minutes at 15400 *g*. The supernatants were stored at −80°C.

The levels of SLPI from vitreous and retinal samples were assessed by Western blot, with each blot being performed in duplicate. The blot was probed with polyclonal antibody against SLPI used for the immunostaining. For the positive control we used serum from rats with *S. aureus* sepsis (data not shown). Fifteen microliters of each homogenate were run under either reducing or nonreducing (r, nr) conditions at ambient temperature using a modified Laemmli method. The samples were electrophoresed on 10–15% polyacrylamide SDS gel at 100 volts for 2 hours and transferred to nitrocellulose membranes at 120 volts for 2 hours (Bio-Rad, Richmond, Calif, USA). The nitrocellulose paper was incubated at room temperature in blocking buffer (PBS, 0.05% Tween 20, 0.5% nonfat dry milk) with the primary antibody (1 : 1000 dilution) and the secondary antibody (1 : 2000 dilution) for 1 hour each on a rotating platform. After three washes with TBS-T (TBS, 0.05% Tween-20), the membranes were incubated in enhanced chemiluminescence solution (Amersham Life Science, Arlington Heights, Ill, USA) followed by exposure to film.

The Western blots from ocular specimens with each one in duplicate were subjected to densitometry analysis (Molecular Dynamics, Sunnyvale, Calif, USA) and normalized to a standard curve to obtain relative SLPI values. 

### 2.4. Statistical analysis

Relative SLPI values were reported as mean ± standard deviation (SD). Statistical analysis comparing the *S. aureus* inoculated, vehicle-injected, and control groups was done using the Mann-Whitney test. A *P* value less than .05 was considered statistically significant.

## 3. RESULTS

**Figure 1:**
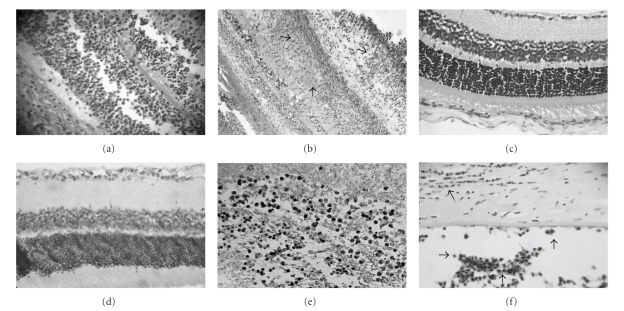
Photomicrographs of immunostaining for SLPI at 24 hours in the endophthalmitis group (panels a-b). Note positive retinal SLPI immunostaining, associated with inflammatory cell infiltration and tissue necrosis. No staining was present in vitreous and retinal structures in the normal and BSS groups (panels c-d). Strong expression of SLPI was identified in endophthalmitis samples at 48 hours at vitreous samples as well as corneal tissue (arrows, panels e-f). (Immunohistochemistry, ×800, panels a-b-e, and ×400 magnification panels c-d-f.)

We used a murine model to elucidate the role of SLPI, an antimicrobial peptide, in endophthalmitis. Immunohistochemical studies show an initial neutrophilic infiltrate consistent with the inflammatory response evident at histopathology examination. At 24 hours after intraocular inoculation with *S. aureus*, there was intense SLPI staining in the vitreous and retinal tissues (Figures 1(a)and1(b)). The eyes from the vehicle-injected and untreated control groups did not show clinical signs of inflammation, inflammatory cell infiltrates, or SLPI staining in the vitreous and retina by immunohistochemistry (Figures 1(c)and1(d)). A few samples (n=3) from the vehicle-injected and control groups showed weak SLPI staining of retinal vessels (not shown). At 24 hours postinoculation, the anterior segment structures did not manifest histological or immunohistochemical changes.

Forty-eight hours post inoculation, eyes from the *S. aureus* inoculated group demonstrated an intense inflammatory reaction at slit lamp examination. In contrast with the eyes 24 hours postinoculation, there was evident inflammation of the anterior segment. The inflammatory infiltrate was more marked with associated tissue necrosis. Immunohistochemically, there was intense and diffuse staining of SLPI in all ocular structures. Positive staining for SLPI in the anterior chamber, corneal epithelium, and stroma corroborated the slit lamp findings of keratitis (Figures 1(e) and 1(f)).

Eyes in the vehicle-injected and control groups did not reveal inflammatory changes at slit lamp examination or in histopathology studies. When examined for presence of SLPI, none of eyes studied had any discernible immunohistochemical reaction.

*S. aureus* infection with its associated inflammatory response is a major trigger for the increased production of SLPI, not observed in the vehicle-injected or untreated control groups at 24 or 48 hours (Table 1).

**Figure 2:**
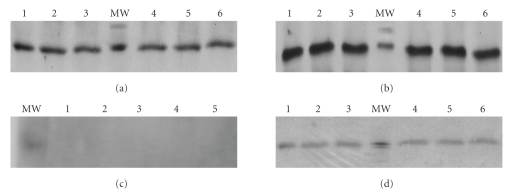
Representative western blots of SLPI expression of supernatant from the endophthalmitis group (a)-(b) and BSS group (c)-(d) were analyzed. Infection with associated inflammation induces the expression of SLPI (12 kDa) at 48 hours in vitreous (a) and retina (b) homogenates in all infected eyes. There is no expression at 48 hours in vitreous samples from injected BSS group (c). However, a weak basal expression at retinal homogenates was found in BSS and control groups (d). Note the strong level of SLPI in the endophthalmitis group (a), (b).

We used Western blots with densitometry to quantify SLPI protein expression in the posterior segment structures. Both the vitreous and retinal homogenates from the *S. aureus* inoculated group at either 24 or 48 hours post injection had high levels of SLPI specific immunoreactivity by Western blot (Figures 2(a)and2(b)). The vehicle-injected and untreated control groups were void of SLPI expression in the vitreous at both time points (Figure 2(c)). In contrast, retinal homogenates from the vehicle-injected and untreated rats showed slight SLPI expression both at 24 or 48 hours postinjection (Figure 2(d)).

The values of SLPI bands from western blots were quantified by densitometric analysis and subjected to statistical analysis (*P* *<* .05 versus control group, Mann-Whitney test) to determine whether there was a difference between the endophthalmitis group and the BSS groups (Table 2).

## 4. DISCUSSION

We hypothesized that SLPI plays a role in the innate immune defense of the eye in response to intraocular inflammation and infection. This study is the first to document that SLPI is strongly expressed in inflamed eyes in an animal model of endophthalmitis and that SLPI expression is directly associated with infiltration by inflammatory cells in ocular tissues. Given what is known about the role of SLPI in other tissues such as lung, skin, and placenta [8, 22, 26], our findings suggest that SLPI is secreted in order to promote the early eradication of invading microorganisms and to protect the eye against proteolytic destruction by inflammatory cells.

We conclude that SLPI expression increases as a result of *S. aureus* infection and the associated inflammatory response. This is supported by our findings that SLPI expression correlates in location, time, and intensity with the clinical infection. SLPI expression and the inflammatory response colocalize in the posterior segment in inoculated eyes. As the infection progresses to the anterior segment at 48 hours, the SLPI immunoreactivity colocalizes to the cornea. By immunohistochemistry, SLPI is upregulated as the mounted inflammatory response intensifies at 48 hours when compared to the 24-hour time point. Thus, SLPI expression also correlates temporally and in intensity with the infectious process.

The absence of elevated SLPI expression in the sham-injected eyes argues against the trauma from the injection as the cause of the SLPI response. The correlation of SLPI in place, time, and intensity with the inflammatory process strongly supports that SLPI is upregulated as a result of the infection and/or inflammation. The weak expression of SLPI in retinal homogenates from control and BSS groups in Western blots may be associated with the retinal vessels that stain for SLPI in immunohistochemistry assays and not be related to production by native retinal cells. The presence of SLPI in the retinal vasculature suggests that SLPI is produced by cells recruited from the extracellular matrix (ECM).

Several microbiological studies describe that the most common pathogens responsible for acute postoperative endophthalmitis are *Staphylococcus* species, the rationale underlying the choice of a clinical isolate of *S. aureus* for our study [1, 3]. The consequences of endophthalmitis are dire: delayed diagnosis and treatment of endophthalmitis may produce irreversible inflammatory damage and may consequently lead to loss of vision [1, 4, 13]. The inflammatory chemotactic factors and toxins from bacteria can induce leukocyte infiltration, principally transendothelial migration of neutrophils from the vascular circulation to the ECM [14]. The ECM serves as a structural scaffold of macromolecules and as a reservoir of inflammatory mediators [15]. The balance between ECM formation and destruction associated with inflammatory and infectious processes is maintained by the presence of endogenous tissue inhibitors of proteases [16, 17].

The concept that certain peptides have antiinflammatory properties and contribute to the innate host defense has been reported in other organ systems [18]. Specifically, SLPI is involved in protection against damage from tissue inflammation [9, 12]. It also neutralizes the action of neutrophil elastase as well as other proteases secreted in the ECM [19, 22]. In addition, SLPI is upregulated in response to proinflammatory cytokines, such as IL-1 and TNF-**α**, and to bacterial products [9, 11]. Recent studies demonstrate that the SLPI is secreted by inflammatory and noninflammatory cells in response to tissue destruction [8, 9, 12, 23–26]. In conjunction with our findings, SLPI likely plays a similarly important pathophysiologic role in bacterial endophthalmitis, Most likely, it is expressed in response to inflammation itself, although our data do not directly rule out SLPI expression directly triggered by bacterial toxins or cell wall components.

In conclusion, we demonstrate that SLPI is not produced in ocular tissues under normal physiologic conditions. This peptide may be secreted in order to promote the early eradication of invading microorganisms and to protect the eye against proteolytic destruction by inflammatory cells. Previous experiments shows SLPI expression in bronchial, nasal, and cervical tissues and in tears [19–21]; our study expands upon these findings and highlights the role of SLPI in intraocular inflammation and ocular innate immunity. The known antiprotease and antimicrobial activities of SLPI suggest that its expression is actively regulated at the site of ocular tissue inflammation. Because of its endogenous antimicrobial activities and role as an inflammatory mediator, further studies addressing the role of SLPI in innate ocular immunity and in wound healing may have consequences in the development of innovative prophylactic and therapeutic strategies for eye disease.

## Figures and Tables

**Table 1:** Immunohistochemical detection of secretory leukocyte protease inhibitor in ocular rat samples.

		*24 h*		*48 h*	
Experimental group		Endoph.	BSS	Endoph.	BSS
Cornea	OD	2/8	0/8	6/8**	0/8
	OS	0/8	0/8	0/8	0/8

Vitreous	OD	8/8**	0/8	8/8**	0/8
	OS	0/8	0/8	0/8	0/8

Retina	OD	8/8**	0/8	8/8**	0/8
	OS	0/8	0/8	0/8	0/8

*Double masked observer determined the total positive immunostained corneas for SLPI in the experimental group. Immunoreactivity is reported as number of positively immunostained eyes/total number of eyes examined in the endophthalmitis group at 24 and 48 hours. **Statistically different when comparing the *Staphylococcus aureus* or *BSS* inoculated eye (OD) to normal control eye (OS). (*P* *<* .05 using Fisher's exact test analysis.) ns = not statistically significant.

**Table 2:** Western blot densitometry analysis of secretory leukocyte protease Inhibitor in ocular rat samples.

		*24 h*		*48 h*	
Experimental group		Endoph.	BSS	Endoph.	BSS
Vitreous	OD	179.37 ± 3.92**	0.25 ± 0.15	191.66 ± 4.12**	0.15 ± 0.17
	OS	0.10 ± 0.28	0.10 ± 0.06	0.23 ± 0.09	0.08 ± 1.14

Retina	OD	195.77 ± 3.26**	10.14 ± 3.04	199.29 ± 1.95**	13.71 ± 2.20
	OS	11.22 ± 2.94	8.72 ± 1.61	11.31 ± 0.11	10.94 ± 1.10

*Double masked observer determined the total positive immunoblots for SLPI in the posterior segment eye structures. The immunoblots for SLPI from endophthalmitis and BSS groups were subjected to densitometry analysis. **Statistically different when comparing the *Staphylococcus aureus* or *BSS* inoculated eye (OD) to normal control eye (OS). *P* *<* .05 using Mann-Whitney test; values are means +/− standard deviation. ns = not statistically significant.
